# Chromatin dynamics and histone modifications in intestinal microbiota-host crosstalk

**DOI:** 10.1016/j.molmet.2019.12.005

**Published:** 2019-12-27

**Authors:** Rachel Fellows, Patrick Varga-Weisz

**Affiliations:** 1 Babraham Institute, Babraham, Cambridge, CB22 3AT, UK; 2 School of Life Sciences, University of Essex, Colchester, CO4 3SQ, UK

**Keywords:** Microbiota, Microbiome, Histone modifications, Acylations, Crotonylation, Chromatin

## Abstract

**Background:**

The microbiota in the human gut are an important component of normal physiology that has co-evolved from the earliest multicellular organisms. Therefore, it is unsurprising that there is intimate crosstalk between the microbial world in the gut and the host. Genome regulation through microbiota-host interactions not only affects the host's immunity, but also metabolic health and resilience against cancer. Chromatin dynamics of the host epithelium involving histone modifications and other facets of the epigenetic machinery play an important role in this process.

**Scope of review:**

This review discusses recent findings relevant to how chromatin dynamics shape the crosstalk between the microbiota and its host, with a special focus on the role of histone modifications.

**Major conclusions:**

Host-microbiome interactions are important evolutionary drivers and are thus expected to be hardwired into and mould the epigenetic machinery in multicellular organisms. Microbial-derived short-chain fatty acids (SCFA) are dominant determinants of microbiome–host interactions, and the inhibition of histone deacetylases (HDACs) by SCFA is a key mechanism in this process. The discovery of alternative histone acylations, such as crotonylation, in addition to the canonical histone acetylation reveals a new layer of complexity in this crosstalk.

## Highlights

• Chromatin dynamics of the host epithelium play an important role in host-microbiota crosstalk.
• Microbiota-derived short chain fatty acids (SCFA) are a dominant determinant in microbiome–host interaction.
• Alternative histone acylations reveal a new layer of complexity in host-microbiota crosstalk.

## 1 The epigenome is shaped by the environment

Each cell in the body of a multicellular eukaryotic organism usually has essentially the same genome in its nucleus, packaged into a highly complex superstructure known as chromatin. The basic building block of chromatin is the nucleosome, composed of eight core histones (H2A, H2B, H3, and H4) around which DNA winds in almost two turns. An additional linker histone H1 “seals off” this structure. Histone tails, normally unstructured but highly conserved peptide components of the histones, protrude from the core nucleosome body and are subject to a plethora of post-translational modifications (PTMs). These various histone PTMs are critical components of gene and genome regulatory mechanisms and are thought to constitute a type of “regulatory language” (the “histone code”), in part by creating binding sites for effector proteins, often called “readers” (reviewed in [[1], [2], [3]]). Histone acetylation is a paradigm histone PTM. This modification occurs on the epsilon amino groups of lysine residues on N-terminal tails of predominantly histones H3 and H4 and is associated with permissive, transcriptionally active chromatin. This modification is mediated by histone acetyltransferases (HATs, “writers”) and reversed by histone deacetylases (HDACs, “erasers”).

Histone lysine methylations are PTMs that have also been well studied, but the functional context is more complex than acetylation. For example, trimethylation of histone H3 at lysine 4 (H3K4me3) is strongly linked to active genes, whereas trimethylation of histone H3 at lysines 9 (H3K9me3) or 27 (H3K27me3) is part of various gene–repressive pathways [4].

The structures of nucleosomes are altered by a plethora of additional proteins of which ATP-dependent nucleosome remodelling factors are an important group (reviewed in [5,6]). These factors can catalyse the eviction or restructuring of nucleosomes, for example by histone dimer eviction or exchange of histone variants. These factors also affect the post-translational modifications of histones, possibly by facilitating these enzymatic steps in a nucleosomal context.

In addition to histones, DNA itself is modified, most commonly methylation of carbon-5 position of cytosines at CpG dinucleotide sequences. Histone and DNA modifications are important components of epigenetic mechanisms that not only allow cells to differentiate into many cell types from one genome blueprint, but also form a part of a cellular “memory” [7]. This “memory” is not only essential for a cell to “remember” its identity, but also constitutes a mechanism by which a cell can integrate external cues, such as environmental influences. Other components of the epigenetic machinery are transcription factor networks and non-coding RNAs, including long non-coding RNAs and microRNAs. Exactly what constitutes an epigenetic mechanism or what should be called “epigenetic” has been the subject of some debate, but we believe that a practical, non-dogmatic approach is useful and we consider everything that moulds the functional output of the genome without changing the underlying DNA sequence to be “epigenetic,” remembering that “epi” stems from Greek for “on top of.”

Human microbiota are very dominant environmental factors that our bodies have to deal with, affecting health and disease. This review discusses recent work investigating how the gut microbiota shapes the epigenome. This is a dynamic and complex field, and there have been a number of recent reviews covering various aspects [[8], [9], [10], [11], [12], [13], [14]]. This report focusses on how this crosstalk shapes the host's genome function through histone modifications, and very recent papers are discussed. As this topic is complex and brings together several fields, a “glossary” box is provided to summarise or explain several critical terms (Table 1).

**Table 1:** Glossary.

Term	Definition and explanation
Acylation	A group of post-translational modifications is produced by covalently adding functional groups to amino acid residues on proteins through acyl linkages. One main type is fatty acylation, the addition of fatty acyl chains to proteins. Acylations include formylation, acetylation, propionylation, crotonylation, butyrylation, hydroxybutyrylation, malonylation, glycosylation, succinylation, benzoylation, and palmitoylation.
AhR	Aryl hydrocarbon receptor is a ligand-activated transcription factor that regulates a variety of cellular processes. Ligand activation causes dissociation from its chaperone HSP90 and binding to aryl hydrocarbon receptor nuclear translocator (ARNT). AhR is an important regulator of immune responses.
Anti-microbial peptides (AMPs)	A diverse group of peptides expressed as part of the innate immune host defence (also called host defence peptides, HDPs). The peptides are usually small (12–50 amino acids) and function, for example, by destabilising the bacterial cell membrane. One group of these peptides is called defensins, which are cysteine-rich cationic peptides. Some defensins are expressed by Paneth cells at the base of the crypts of the small intestine.
Bromodomain	The bromodomain is a protein motif that is conserved in eukaryotes and found in over 100 proteins. It preferentially binds acetylated lysine residues such as those found on histones.
Commensal bacteria	These bacteria are part of the microbiota, for example, in the gut. They do not hurt the host, but also do not provide significant benefits.
Conventionalised mouse	A mouse that was initially germ-free (see below) but has been re-colonised with normal microbiota.
Epigenetics	The study of heritable phenotypic changes in gene expression without changing the underlying DNA sequence. Derived from the Greek “epi” meaning “on” or “above.” This term is often used to describe many DNA and chromatin-associated modifications.
Gastrointestinal tract	An organ system that takes in, digests, and absorbs nutrients along with the removal of waste products. It comprises the mouth, oesophagus, stomach, small intestine (duodenum, ileum, and jejunum), caecum (and attached appendix), colon, rectum, and anal canal.
Germ-free mouse	Germ-free animals have no microorganisms living in or on them. The generation and maintenance of germ-free mice is a challenging task. Germ-free mice are bred in isolators that block exposure to microorganisms, keeping them free of detectable bacteria, viruses, and eukaryotic microbes. Re-colonising these mice with defined microorganisms generates gnotobiotic mice. An alternative to using germ-free mice is treating mice with a cocktail of antibiotics to get rid of a majority of bacteria [22].
GPCRs	G protein-coupled receptors are a large family of membrane proteins that bind a specific molecule on the extracellular side and couple to a signalling response on the intracellular side. Ligand binding triggers a conformational change that activates the alpha subunit of the G protein, which releases the gamma and beta subunits to generate further signalling reactions in the cell to elicit a response.
HDAC	Histone deacetylase. HDACs should really be called lysine deacetylases (KDACs) as they also deacetylate proteins other than histones. Based on sequence homology, 18 human HDACs are grouped into four classes. Class I enzymes are comprised of HDAC1, 2, 3, and 8. Class II enzymes are composed of HDAC4, 5, 6, 7, 9, and 10. Class III enzymes consist of seven sirtuins that are NAD-dependent protein deacetylases and/or ADP ribosylases. Class IV contains only HDAC11, which shares sequences similar to both class I and II proteins. Several inhibitors against HDACs have been developed with promise in cancer therapy [88].
Haemolymph	The equivalent of blood in vertebrates, haemolymph is a fluid that circulates around the interior of arthropod bodies as part of the open circulatory system to exchange materials with tissues. Arthropods include *Drosophila melanogaster*, which is used frequently as a model organism in biological research.
Histone code	The histone code hypothesis was formulated to express the idea that histone modifications, including combinations of these modifications, regulate DNA-templated processes, such as transcription [89]. Furthermore, histone modifications are thought to act, at least in part, by creating binding platforms for effector proteins, such as nucleosome remodelling factors.
IECs	Intestinal epithelial cells line the gut lumen and form the first line of defence after the mucus layer barrier (see Figure 1). Stem cells in the crypt base generate Paneth cells, label-retaining cells, transit-amplifying cells, enterocytes, enteroendocrine cells, tuft cells, and goblet cells required to maintain the epithelial niche. IECs are supported by the lamina propria.
IELs	Intestinal epithelial lymphocytes are T lymphocytes derived from naïve T cells in the thymus and are present in the epithelial and lamina propria layers of the intestine. Upon detection of antigens, they release cytokines to kill infected cells.
Inflammatory bowel diseases	Chronic disorders of the digestive tract associated with prolonged inflammation. Two main types are ulcerative colitis, which occurs in the colon, and Crohn's disease, which can occur anywhere along the gastrointestinal tract.
MAMPs	Microbial (or pathogen)-associated molecular patterns are motifs of microbial-specific structures that elicit a host response. They include flagellin, lipopolysaccharide, peptidoglycan, and viral single-stranded RNA.
Microbial dysbiosis	An imbalance in the microbiota associated with overrepresentation of certain microbial species caused by antibiotic use, poor diet, or chronic stress. There is insufficient evidence as to whether microbial dysbiosis is a direct cause of inflammatory diseases or a result of them. As microbial species are highly variable between individuals, determining when the microbiota is in dysbiosis can be difficult. A narrower definition describes microbial imbalance that causes disease in line with Koch's postulates (criteria for establishing a causal relationship between a microbe and disease).
Microbiome	This term is sometimes used synonymously with microbiota. However, a narrower definition is “the collective genomes of the microbiota in or on an organism.” The microbial genome typically has 100 times more genes than the host genome. Major phyla of the human bacterial gut microbiome are Firmicutes, Bacteroidetes, Actinobacteria, and Proteobacteria.
Microbiota	The community of microorganisms (bacteria, archaea, and fungi such as yeasts, protozoa, viruses, and phages) found in and on a multicellular organism. These microorganisms may be symbionts, commensal, or pathogenic. The word “microbiota” is a plural term (singular would be “microbiotum”) similar to the term “people.”
Nucleosome	The basic unit of DNA packaging consisting of an octamer of H2A, H2B, H3, and H4 histones that coil approximately 146 base pairs of DNA.
Obesogenic diet	A high-fat diet given to mice to induce obesity.
PRRs	Pattern recognition receptors are key elements of the innate immune system. Receptors identify bacterial signals to enable responses to pathogenic bacteria. PRRs include Toll-like and nucleotide binding oligomerisation domain (NOD)-like, C-type lectin, and RIG-1-like receptors.
PTM	Post-translational modification. Chemical modification of amino acid residues after their assembly into a protein during translation by the ribosome using an mRNA template. This can alter the chemical properties of the protein or change interactions with other proteins. PTMs include acetylation, phosphorylation, hydroxylation, glycosylation, lipidation, ubiquitination, or deamidation.
SCFA	Short-chain fatty acid(s). A carboxylic acid less than six carbons in length. The predominant SCFA in the intestine are acetate (C2), propionate (C3), and butyrate (C4). Other SCFA include formate (C1), crotonate (C4), isobutyrate (C4), valerate (C5), and isovalerate (C5).
SOPF	Specific or pathogen-free. Laboratory organisms free from certain infectious agents that are capable of pathogenicity or may interfere with an experiment.
Westernised diet	A high-fat, high-salt diet given to laboratory mice to replicate a “typical” diet consumed in developed countries.
Xenobiotics	A chemical compound not normally produced or consumed by an organism. Foreign compounds can be drugs, carcinogens, or pesticides.
YEATS domain	Named after the domain containing Yaf9, ENL, AF9, Taf14, and Sas5 proteins, the YEATS domain is a protein motif that preferentially binds crotonylated lysine residues. This domain has been linked to chromatin structure and gene expression.

## 2 The microbial world in humans

The world is permeated, if not dominated, by microbes, and microbes thrive in the most hostile environments on Earth. Thus, it is unsurprising that our bodies are also home to a staggering number and diversity of microbes, including bacteria, archaea, protists, yeasts, and viruses. Technological developments, especially next-generation sequencing-based metagenomics methods, have revolutionised our understanding of the microbial world, including human microbiota. We have learned that complex ecosystems of microbes cover many mucosal surfaces of the human body, such as the skin, gut, vagina, lungs, uterus, and bladder [[15], [16], [17], [18]].

The microbiota and host have coevolved from the earliest multicellular organisms onward and it has been argued that pressure on the host to control the microbiota has been an important evolutionary driver [19]. Thus, the host microbiome has been termed “an ecosystem on a leash” [19]. As Foster et al. wrote: “Host control over the microbes (as opposed to microbial control of the host) can be predicted, because there is only one host in the interaction, in contrast to the myriad microbes. Thus, unlike individual microbes, a host can easily influence the entire microbiome, and benefit from doing so” [19]. Therefore, while we will present evidence in this review that the microbiota manipulates the epigenetic machinery for interaction with the host, we can expect that this interaction also shaped the epigenetic machinery during evolution.

**Figure 1:**
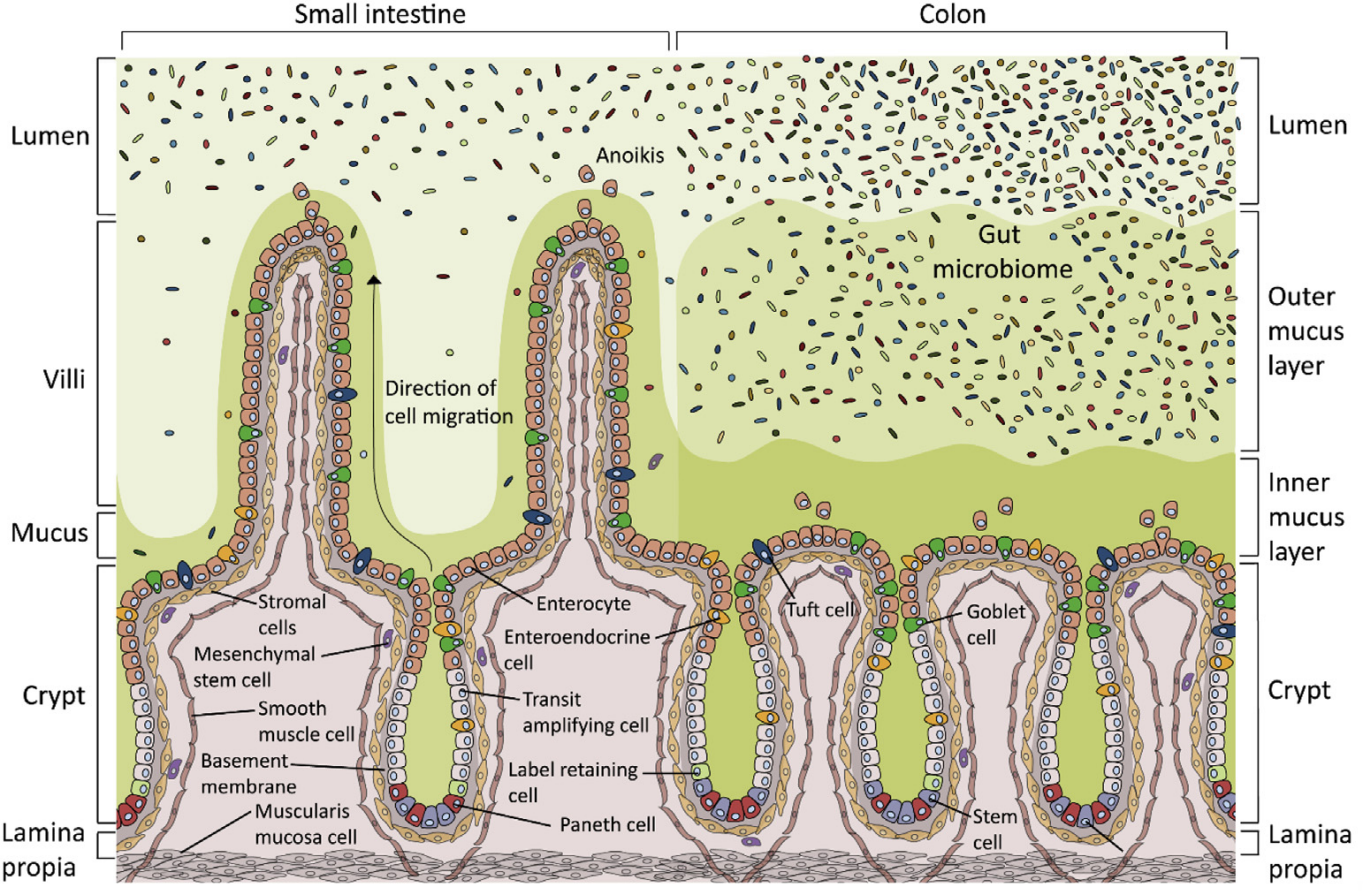
**The structure of the small intestine and colon epithelium.** The intestine has a large surface area to enable efficient absorption of dietary nutrients. It is comprised of pocket-like crypts containing stem cells that generate all of the necessary cell types for the intestinal epithelium. Cells develop as they move up the crypt walls before being lost by anoikis (apoptosis induced by loss of cell contact) into the gut lumen. In the small intestine, cells are lost at the top of the villi, which are finger-like projections that further increase the surface area. There are many cell types in the intestine, and the absorptive enterocytes and mucus-secreting goblet cells are the most abundant. Transit-amplifying cells are proliferative and lineage committed to become enterocytes. Enteroendocrine cells secrete hormones, tuft cells secrete prostanoids and opioids, and Paneth cells secrete anti-microbial peptides and support the stem cells. Label-retaining cells are quiescent Paneth cell precursors [90]. The small intestine contains a single diffuse layer of mucus that is not attached to the epithelium and contains some bacteria. The colon contains inner and outer mucus layers. The inner mucus layer is compact and attached to the epithelium and is normally free from bacteria. The outer mucus layer is diffuse with an undefined border and provides a habitat for intestinal bacteria. The colon microbiota is larger and more diverse than that of the small intestine [91]. The lamina propria is a thin layer of connective tissue that supports the epithelial cell niche. Intestinal-associated immune cells, lymphatic vessels, and capillaries are not shown. The muscularis mucosae, a thin layer of muscle, separates the lamina propria from the underlying submucosa (not shown). The epithelium, lamina propria, and muscularis mucosa together make up the mucosal layer [92].

In many mammals, including humans, the greatest number of microbes are found in the colon (Figure 1). It is estimated that the number of microbes in the colon at least matches the total number of host cells in a human [20]. The microbiota create a complex ecosystem where several species compete with, depend on, or influence each other. Importantly, the microbial community in the colon is highly diverse with at least ∼1000 different species. Despite some redundancy in function between species, this means that the combined microbial “genome” is more than 100-fold greater than that of the host. This has important implications for the host, as the microbiota contain unique genes that are absent in the host's genome. Many of these genes encode enzymes that break down dietary components, such as complex carbohydrates, and make these absorbable and available to the host. In this way, the microbiota make an important contribution to the host's extraction of nutrients and energy from the diet [21]. This can be seen in germ-free mice that are usually leaner then their microbiota-containing counterparts [22]. In addition to helping in the digestion of food, bacteria also synthesise essential vitamins and are key in training the immune system. Furthermore, our normal commensal microbiota protect us from pathogenic microbes, in part by simply competing them out of space and nutrients. Thus, the microbiota exert an important and largely beneficial role in human life. In their role in digesting food and generating vitamins, the microbiota could be considered almost an organ in the human body. This notion is strengthened if one considers that structures in the gut, such as the caecum, evolved to house the microbiota. This would be a highly dynamic organ, not only changing dramatically in size depending on food intake and digestive status, but also in the species composition of microbes. In fact, the microbial composition differs from person to person because it is strongly affected by nutrition, lifestyle, and other factors [23,24].

Furthermore, the microbiome composition evolves over the human lifetime, from its acquisition during and after birth, maturing after weaning, and changing even into old age [24]. However, the microbiota can turn into the enemy within us. Not only can humans ingest harmful bacteria, such as *Salmonella* that invade and poison our body [25], but the body can also overreact to the presence of the gut microbiota, for example, as a result of genetic predisposition. This can lead to inflammatory bowel diseases (IBD), such as Crohn's disease and ulcerative colitis [26]. Furthermore, the microbiota have been identified as contributing factors in cancer processes, especially gastric and colon cancers. The role of *Helicobacter pylori* in gastric cancer is an example [27].

In summary, the microbiome is a dominant force in our lives, and understanding how microbiota-host interactions are regulated is important.

## 3 Microbiota-host crosstalk through microbial metabolites

**Figure 2:**
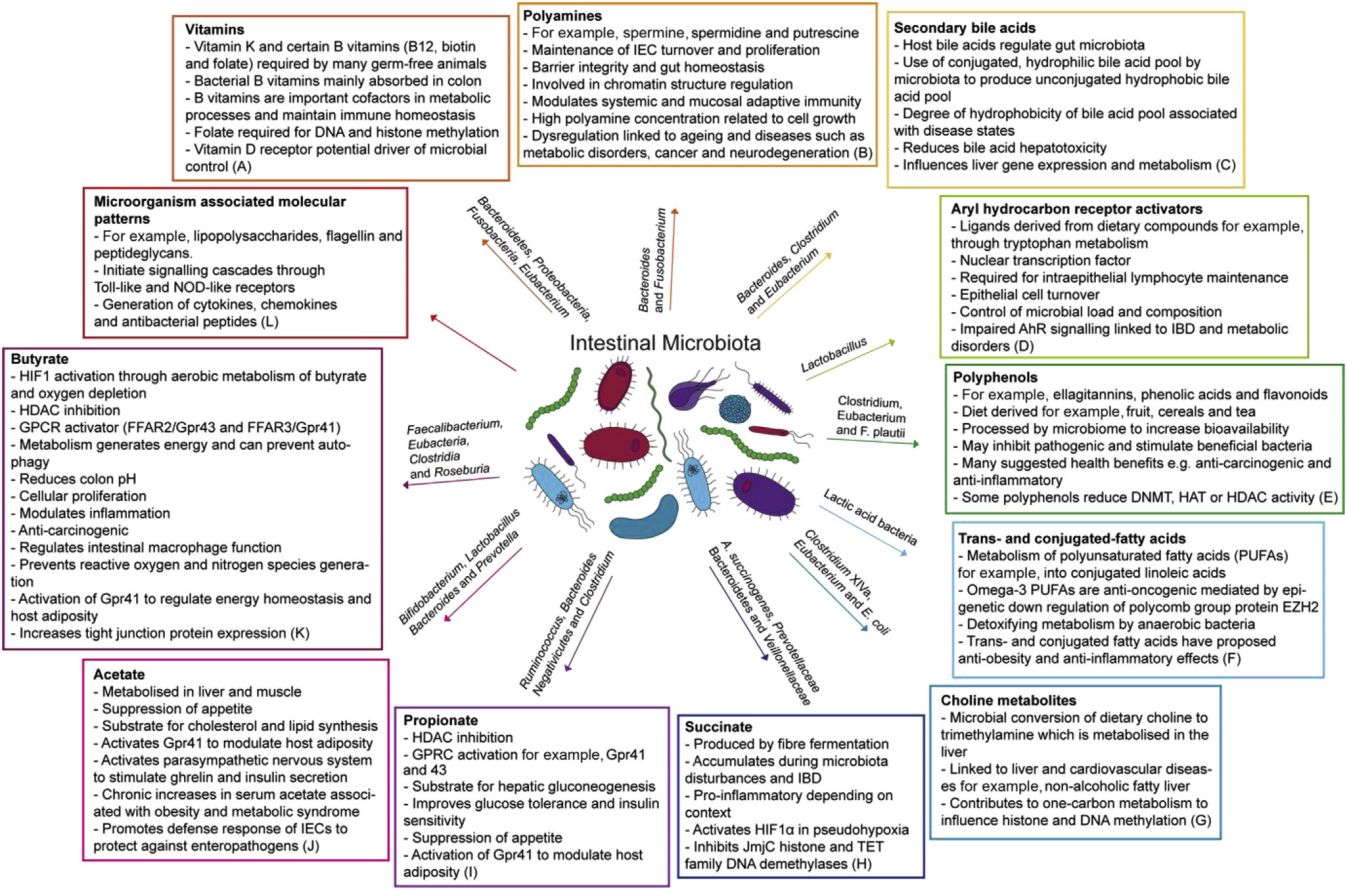
**Microbial metabolites influence host function.** A non-exhaustive list of microbial generated molecules and their effects on cellular and organismal function. Some of the bacteria species that generate the specified metabolites are listed on the arrows. References for **(A**) [34,35,[93], [94], [95], [96], [97], [98]], **(B)** [36,99], **(C)** [100,101], **(D**) [30,102], **(E)** [[103], [104], [105], [106], [107], [108]], **(F)** [[109], [110], [111], [112], [113], [114]], **(G)** [115,116], **(H)** [99,[117], [118], [119], [120], [121]], **(I)** [[122], [123], [124], [125]], **(J)** [122,123,[126], [127], [128]], **(K)** [37,38,[40], [41], [42],[122], [123], [124],^129^,[129], [129], [130], [131], [132], [133]], and **(L)** [28,29,134].

The crosstalk between the microbiota and host occurs through a large variety of molecules, such as bacterial structural components and metabolites. Bacterial cell wall components or flagellar proteins are recognised by the host's cells through specific receptors (so-called pattern recognition receptors, PRRs) in innate immune responses. Toll-like receptors are well-studied PRRs. The microorganism-associated molecular patterns (MAMPs) include lipopolysaccharides, flagellin, and peptidoglycans. These initiate signalling cascades, for example, leading to an anti-bacterial response through the generation of cytokines, chemokines, and/or anti-bacterial peptides (reviewed in [28,29]). Another important mechanism by which the microbiota interact with the host is through the generation of bioactive molecules that are taken up in the host's cells and affect cellular functions, especially gene regulation [29]. Several key metabolites have been studied in this context, including short-chain fatty acids (SCFA), polyamines, vitamins, and aryl hydrocarbon receptor (AhR) ligands. Figure 2 summarises some of these bacterially derived molecules and their impact on the host.

The AhR is a nuclear receptor type of transcription factor that is activated by binding to diverse ligands, including xenobiotics, plant or bacterial metabolites, or bacterial pigments [[30], [31], [32]]. AhR function has been shown to be required for intestinal immunity in mice by maintaining intestinal intraepithelial lymphocytes [30].

Bacteria synthesise several vitamins, such as B12 (cobalamin), riboflavins, and folate [33]. As folate is required for DNA and histone methylation, the commensal bacteria have a potentially broad impact on epigenetic mechanisms [34,35].

Polyamines (PA), such as spermine, spermidine, and putrescine, are essential for life in eukaryotes and prokaryotes and are involved in many processes, such as gene expression, chromatin structure regulation, stress response, differentiation, and proliferation (for review [36]). Normally, PA are derived from the diet and absorbed by the small intestine, but can also be generated by the microbiota in considerable amounts in the colon, where they are thought to support epithelium health [36]. How microbial PA affect the host's chromatin is poorly understood.

SCFA constitute a major class of bacterial metabolites. They are generated by the microbiota through the fermentation of complex carbohydrates as a metabolic waste product in the colon (and in the caecum in many animals) in large amounts and have a profound impact on the host's physiology (reviewed in [37]). The major microbial-derived SCFA are acetate, propionate, and butyrate. Estimates of SCFA concentrations vary between studies and different diets. Rombeau et al. approximated SCFA concentrations in the content of the human colon to be 75 m*M* for acetate, 30 m*M* for propionate, and 20 m*M* for butyrate [38]. These SCFA are generated by several bacterial species and there is cross-feeding between bacterial species; for example, acetate- and lactate-producing *Bifidobacterium* species have been shown to feed the butyrate-producing *Faecalibacterium prausnitzii* [39].

While acetate and propionate are released into the bloodstream through the portal vein, butyrate is mostly absorbed and metabolised by the colon epithelium, which constitutes the preferred energy source in this tissue [21]. In fact, the absence of microbiota in germ-free mice and, therefore, the lack of SCFA causes a complete remodelling of metabolism in the colon epithelium with a dramatic upregulation of autophagy to compensate for the loss of microbial SCFA [21]. Antibiotic treatment to deplete microbiome confirms the importance of the microbiota in energy generation and metabolism [40]. The oxidation of butyrate in the epithelium affects O_2_ levels, causing activation of the oxygen sensor HIF1, which in turn affects the response to pathogens [41,42]. Butyrate inhibits cellular proliferation of intestinal stem/progenitor cells at physiologic concentrations and it has been suggested that the epithelial cellular anatomy reflects this influence, protecting the stem and proliferating cells from the effects of butyrate by sequestering them in crypts [43]. Thus, butyrate has different effects on cells depending on their location along the crypt axis, with stem cell niche being relatively depleted of butyrate while villus cells use butyrate as a principal carbon source [43].

Butyrate and propionate are effective HDAC inhibitors at the concentrations that are generated in the colon and this constitutes an important mechanism by which these SCFA affect physiology. SCFA also activate G protein-coupled receptors (GPCRs, also called free fatty acid receptors, FFARs). GPR43 and 41 have been studied in this respect. In both capacities, as HDAC inhibitors and activators of GCPRs, the bacterial-derived SCFA suppress inflammatory responses (reviewed in [37]). SCFA might also promote histone modifications by metabolic conversion to acetyl-CoA and other SCFA-CoA precursors to be transferred to histones by HATS such as p300/CBP (see below [44,45]).

## 4 Histone modification in microbiota-host crosstalk

It has been known for decades that there is a link between dietary fibre content, production of SCFA by the microbiota, and histone acetylation in the gut [46]. A recent study examined the effect of the microbiota and diet on histone modifications using mass spectrometry analysis [47]. The researchers employed conventionally raised, germ-free, and microbiota re-colonised (“conventionalised”) mice to address the role of the microbiota [47]. Because conventionally raised animals exhibit developmental differences vs their germ-free controls (reviewed in [48]), the use of the conventionalised mice enabled exploration of effects related to the presence or absence of the microbiota. This study is important as it showed that the gut microbiota effected histone acetylation and methylation not only in the colon, but also in the liver and white adipose tissue and that generation of SCFA by the microbiota is a dominant driver of this process. The researchers found that the presence of microbiota robustly promoted histone acetylation of H3 and H4 at multiple lysine residues in various tissues, while changes in H3 methylation were subtle but still significant [47]. Some histone PTMs appeared to be similarly regulated across all tissues surveyed, while other changes were tissue specific. Interestingly, feeding mice a diet high in fat and sucrose and low in fermentable complex carbohydrates (HF/HS-diet, “Western-style diet”) suppressed microbiota-driven SCFA production and chromatin effects observed in a fibre-rich diet. HF/HS-fed conventionally raised mice displayed higher hepatic total cholesterol and triglycerides vs diet-matched germ-free controls and chow-fed mice, showing that HF/HS feeding impacted the host's metabolic state in a microbiota-dependent manner. The presence of microbiota and the diets manifested themselves in gene expression in the liver and affected many genes related to metabolism.

Gut microbiota alter the expression of genes linked to metabolites that are required for histone PTMs. For example, expression of ATP citrate lyase (Acly), an enzyme essential for glucose-driven, but not acetate-driven, histone acetylation in mammalian cells [49], was decreased in conventionally raised vs germ-free mice under both chow and HF/HS feeding [47]. This suggested that the presence of bacterial SCFA or lipids from HF/HS feeding may suppress glucose-driven histone modification. The authors did not examine how changes in histone modifications, for example, over promoters, are linked to changes in gene expression, such as by ChIP-seq. Overall, this study highlights the intimate link between diet, the microbiota, and genome regulation in the whole organism.

## 5 Alternative histone acylations in microbiota-host crosstalk

Progress in the analysis of histone PTMs by mass spectrometry has allowed the identification of a range of new modifications, many of which can be summarised as alternative acylations. These include histone crotonylation, butyrylation, hydroxybutyrylation, and propionylation (reviewed in [[50], [51], [52]], see Table 2for a summary). These modifications are also linked to metabolic pathways. For example, histone crotonylation is promoted by the addition of crotonic acid to cell culture media, as crotonic acid is converted to crotonyl-CoA by the enzyme ASCC2 [53]. Histone crotonylation changes the functionality of nucleosomes compared to histone acetylation as it creates specific binding platforms for YEATS domain containing chromatin remodelling factors. Although both modifications are associated with active chromatin, crotonylation promoted gene expression to a greater extent than acetylation in a cell-free assay [[54], [55], [56]].

**Table 2:** Histone acylations and their “writers,” “readers,” and “erasers”.

Modification	Structure	Writer	Reader	Eraser
Acetylation		p300 (CBP and p300), MYST (Tip60, MOF, MOZ, and HBO1), and GCN5 (GCN5 and PCAF) (a)	Bromodomain (BRD2, BRD9, TAF1, and CECR2), PHD (MOZ and DPF2), and YEATS (AF9 and YEATS2) (b)	Zn^2^⁺-dependent (HDAC1-11) and NAD⁺-dependent (SIRT1-7) (c)
Propionylation		p300/CBP, PCAF, GCN5, MOF, HBO1, and MOZ (d)	Most BRDs (CECR2, BRD2-4, 7, and 9, and TAF1), MOZ, DPF2, and AF9 YEATS2 (e)	SIRT1/2/3 (f)
Butyrylation		p300/CBP, PCAF, and GCN5 (g)	TAF1 (2), BRD7, BRD9, CECR2, MOZ, DPF2, and AF9 YEATS2 (h)	SIRT1/2/3 (i)
Crotonylation		p300/CBP and MOF (j)	TAF1 (2), AF9, YEATS2, MOZ, and DPF2 (k)	HDAC1-3 and SIRT1/2/3 (l)
β-hydroxybutyrylation		p300/CBP (m)	MOZ and DPF2 (n)	HDAC1-3 and SIRT3 (o)

**Histone acylations and their modifying enzymes**. Histone acylations are set down by “writers,” acyltransferases, bound by “readers” for downstream events, and removed by “erasers,” deacylases. References: (a) [135], (b) [[136], [137], [138]], (c) [139], (d) [51,[140], [141], [142], [143], [144]], (e) [145,146], (f) [147], (g) [51,140,141], (h) [50,55,56,145,146], (i) [147], (j) [148,149], (k) [50,[54], [55], [56],^58^,^145^,^146^,^150^], (l) [[57], [58], [59],^151^,^152^], (m) [144], (n) [146], and (o) [52,56,67].

**Figure 3:**
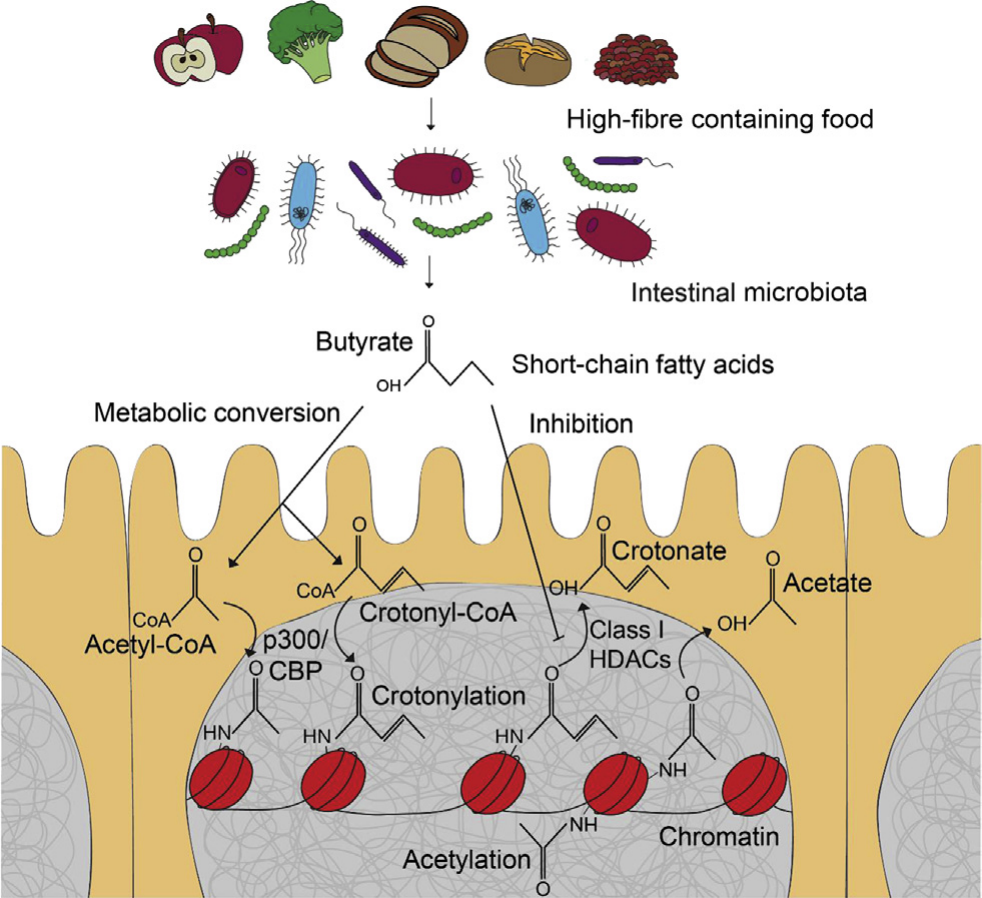
**Current model of how microbial-derived SCFA affect histone acetylation and crotonylation.** The intestinal microbiota digests fibre present in dietary components, such as apples and brown bread, into SCFA. Butyrate is the main SCFA taken up by intestinal epithelial cells. Butyrate inhibits class I HDACs to reduce the removal of acetylation and crotonylation from the histone. It might also promote histone crotonylation and acetylation by metabolic conversion to acetyl-CoA and crotonyl-CoA precursors to be transferred to histones by p300/CBP.

We used mass spectrometry to canvas PTMs, including crotonylation, in histones isolated from the intestinal epithelium [57]. We found that histone crotonylation is a relatively abundant modification in the intestinal epithelium (and the brain), with H3K18cr identified as the most prevalent crotonylation. When we acutely depleted microbiota in mice with a 3-day course of a cocktail of antibiotics, this not only reduced luminal SCFA, but significantly affected global histone crotonylation levels in the gut. We showed that butyrate acted as a histone decrotonylase inhibitor and found, consistent with several other studies published around that time [58,59], that class I HDACs are potent histone decrotonylases [57]. Therefore, our study emphasises the inhibition of HDACs through SCFA, especially butyrate, as an important mechanism for microbiota-host crosstalk. Similar to that observed in other cell types, we found that H3K18cr “peaks” over promoter regions of many genes and its level seems to correlate with gene expression [53,57]. Interestingly, many of the genes with higher levels of crotonylation over their promoters have been linked to cancer pathways. More recently, we found that promoter chromatin crotonylation reflects gene expression changes dependent on microbiota (Fellows et al. in revision). Thus, it appears that promoter crotonylation is an important mechanism for microbiota-host crosstalk in the gut. Our current model of how bacterial-derived SCFA affect histone crotonylation is shown in Figure 3.

## 6 HDACs in microbe-host interactions

The previous sections highlighted the importance of HDACs in microbiota-host crosstalk, mainly because the microbial-derived butyric and propionic acids are HDAC inhibitors. Thus, it is unsurprising that HDACs were found to play a critical role in microbiota-host crosstalk. This is illustrated with HDAC3 in a study from the Artis Lab [60]. Intestinal epithelium specific deletion of HDAC3 (HDAC3^ΔIEC^) led to gene expression and corresponding H3K9ac level changes in affected genes and a progressive loss of Paneth cells, with evidence of Paneth cell death [60]. Paneth cells are found at the base of the small intestinal crypt, where they play a role in regulating microbiota-host interactions by secreting anti-bacterial peptides (see Figure 1 [61]). Thus, consistent with the loss of Paneth cells, the HDAC3^ΔIEC^ mice exhibited increased translocation of bacteria through the epithelium and increased intestinal inflammation, as well as increased susceptibility to oral *Listeria monocytogenes* infection. Remarkably, Paneth cell viability was not affected in HDAC3^ΔIEC^ mice raised under germ-free conditions, and alterations in the majority of HDAC3-dependent transcriptional pathways, including those involved in anti-microbial defence, were not seen. Thus, it appears that HDAC3 is required to respond to bacterial cues and translates this to a gene expression programme that protects intestinal integrity. A follow-up study from the Alenghat Lab demonstrated that HDAC3 mediates communication between intestinal epithelial cells and resident lymphocytes, thereby promoting resistance against infection by pathogenic microbes [62]. Whether these HDAC3 actions occur through deacetylation of histones or other factors or an enzymatic-independent role of HDAC3 remains to be discovered. It will be exciting to determine the bacterial cues involved in these pathways.

Sirt1 belongs to the class III group of NAD + dependent deacetylases, also called sirtuins. Several sirtuins deacetylate histones, but they also have other targets. Epithelial deletion of Sirt1 led to age-dependent enhanced inflammation in one study [63], while another reported protection against colitis and enhanced anti-bacterial defence in the intestine [64]. Both studies reported changes in the microbiota upon Sirt1 deletion. Whether chromatin deacetylation is involved in these processes remains to be elucidated. Deacetylation of transcription factor SPDEF was implicated in the observed activity of Sirt1 in the intestine [64].

Sirt2 is another class III deacetylase/sirtuin. Studies of this enzyme in cultured human cell lines (epithelial cervical adenocarcinoma cell line HeLa and colorectal adenocarcinoma cell line Caco-2) and mouse spleen tissue showed that this enzyme plays a critical role in the pathogenic infection of cells by *L. monocytogenes* [65]. Sirt2 is normally predominantly cytosolic, but upon infection by *L. monocytogenes*, it translocates to the nucleus to tightly bind to chromatin and deacetylate H3K18ac. This in turn leads to the repression of genes normally involved in limiting infection [65,66]. These findings highlight that (1) H3K18 is a potentially critical residue in host–pathogen interactions, (2) a histone modifier is essential for infection by a pathogen, and (3) bacteria can subvert the host's biochemistry for their own purposes. Overall, the previously described studies demonstrate the importance of histone deacetylation in host-microbe crosstalk. Future studies will need to address to what extent histone deacylation processes, such as decrotonylation, are important in this crosstalk, as many HDACs can remove other acyl groups from histones, such as HDAC1-3 acting as a decrotonylase and SIRT3 as a dehydroxybutyrylase (see Table 2 [[57], [58], [59],^67^]).

## 7 Microbiota affect histone modifications over regulatory elements in conjunction with diet

Several histone modifications are linked to regulatory elements, such as promoters and enhancers. For example, H3K27ac in combination with H3K4me1 is often found over active enhancers, while H3K4me1 without H3K27ac marks poised enhancers. Therefore, such histone modification combinations are used to identify candidate enhancer elements [68]. A study from the Wade Lab examined how microbiota in combination with diet affected H3K27ac and H3K4me1 genome-wide using ChIP-seq in colon epithelial cells in a mouse model [69]. Consistent with previous work, they found that an obesogenic diet (high-fat diet, HFD) markedly altered the gut microbiota. This, in turn, caused a reduction in microbial-derived butyrate and changes in mouse metabolic physiology. Their findings showed that the gut microbiota in combination with an obesogenic diet changed the enhancer landscape with respect to these modifications and also affected binding of a critical transcription factor in host-microbiota crosstalk, HFN4alpha, along with concomitant changes in gene expression. They also found that many of these changes were similar to those seen in the colon cancer process. Remarkably, transplantation of the bacteria from HFD-fed, but not from control diet-fed mice, into germ-free mice led to recapitulation of the HFD-associated epigenetic changes. This work demonstrates how an obesogenic diet, in combination with microbiota, may impact disease risk, potentially predisposing to cancer by activating pathways similar to those found in cancer cells. The authors speculated that HFD microbiota is involved in generating metabolites from the HFD that lead to an epigenetic reprogramming of the enhancer landscape, illustrating the complexity of microbiota–diet–host interactions [69].

## 8 Epigenetics and IBD: histone H3K4me3 changes link IBD to microbiota-host interactions

In general, the causes of IBD are complex, involving environmental triggers and genetic susceptibility of the host [26]. Aberrant microbiota-host interactions are prime candidates driving IBD and it is important to understand to what extent epigenetic pathways underlie these defective responses. Alterations in DNA methylation have already been linked to IBD [[70], [71], [72], [73], [74]], but what about other epigenetic features? A recent study mapped genes that showed changes in the histone modification H3K4me3 in intestinal epithelial cells from terminal ilea of newly diagnosed paediatric Crohn's disease (CD) patients and compared these findings with changes in gene expression [75]. Remarkably, the changes in H3K4me3 seemed to identify the CD patients more robustly than the changes in gene expression. The researchers compared these changes with those seen in H3K4me3 in ileal epithelial cells between germ-free mice and conventionally housed mice. These global analyses showed that the presence of microbiota in the gut resulted in many changes in H3K4me3 in IECs. This further demonstrated that a significant proportion of the loci identified in the patients exhibited changes in the mice dependent on the presence of the microbiota, identifying an “epigenetic profile of IBD that can be primed by commensal microbes” [75]. The patient sample in this work was relatively small, and thus, it would be very interesting to see this type of study expanded with more patients, possibly with different forms of IBD. This study sheds new light onto pathways by which microbiota might predispose to intestinal inflammation and illustrates how epigenetic analyses can complement other approaches for identification of epithelial abnormalities.

## 9 Demethylase KDM5 and the microbiota in the gut–brain axis

There is tantalising evidence that suggests a role of the gut microbiota in intellectual disability (ID) and autism spectrum disorder diseases (ASD). Genome-wide association and family studies have implicated several chromatin remodelling factors and histone modifiers in these diseases, including members of the KDM5 family of demethylases that remove histone H3K4 methyl groups. A group of researchers took advantage of the fact that that *Drosophila* has only one KDM5 paralog (humans have four KDM5 paralogues) and has a relatively simple microbiome to examine the role of KDM5 in intellectual deficiency and autism spectrum disorder behaviour models in the fly [76]. They found that reduced levels of KDM5 in a fly *kdm5* mutant caused a global increase in H3K4me3 in the gut concomitant with intestinal barrier disruption, making the gut permeable to microbes. This was accompanied by a change in the gut microbiota, including a reduction in *Lactobacillus plantarum L168* and impaired fly social behaviour. These changes were not observed in flies reared germ-free or after antibiotic treatment. Probiotic treatment of mutant flies with *L. plantarum L168* restored intestinal barrier function and improved social behaviour toward normal. Together, these findings indicate that ablation of KDM5 causes a change in behaviour, at least in part by altering the gut microbiota. Furthermore, the reported activities of KDM5 depended on its demethylase activity, and the researchers implicated the misregulation of innate immunity genes to an aberrant increase in H3K4me3 over their promoters. While this study did not rule out that a non-histone target is critical in the described functions of KDM5, it is likely that chromatin regulation plays an important role in the process. It is not yet clear exactly how the misregulation of the microbiota on KDM5 mutation affects social behaviour. However, the researchers implicated an increase in the neurotransmitter serotonin, which may be microbiota dependent. Interestingly, another study identified histone serotonylation in combination with methylation (H3K4me3Q5ser) as a new histone PTM linked to active genes [77]. This new modification was found to be most abundant in the brain and gut. Whether there is a link between microbiota and histone serotonylation remains to be investigated. In summary, research on KDM5-microbiota interactions is an exciting illustration of how chromatin dynamics links microbiota to the physiology of tissues far from the gut, raising the question if manipulation of the gut microbiota can ameliorate ID and ASD in humans.

## 10 ATP-dependent chromatin remodelling factor CHD1 and host–microbiome interactions in *Drosophila*

*Drosophila* with its relatively simple microbiome also provided insights into the role in host–microbiome interactions of a member of another important class of chromatin factors, ATP-dependent nucleosome remodelling factors, CHD1, which is required for the replication-independent incorporation of histone H3 variant H3.3 into chromatin [78]. Following the observation that deletion of this factor led to misregulation of genes involved in immune responses, stress responses, and detoxification in larvae, Alexandra Lusser et al. found that the loss of CHD1 led to the increased expression of anti-microbial peptides (AMP) in the gut. However, it also rendered flies susceptible to infection by the bacterium *Pseudomonas aeruginosa* upon ingestion of the bacteria [79]. They found that the bacterial load was significantly elevated in *Chd1* mutant flies in the gut and fly body outside the gut after oral infection. This suggested that the gut epithelium was much more permissible to the passage *of P. aeruginosa* and possibly other bacteria into the haemolymph, causing the flies to die. These findings suggest that a misbalance of expressed AMP and other immune factors may lead to dysbiosis and thus susceptibility to the *P. aeruginosa* infection. To substantiate this further, the group performed microbiome analysis using 16S rRNA sequencing [80]. This showed a loss of species diversity in mutant flies. For example, on the family level, the bacterial community in the wild-type flies’ guts of *Pseudomonadacea*e, *Enterobacteriaceae*, *Comamonadaceae*, and *Staphylococcaceae* together comprised ∼19% of the fly microbiota, but these families were nearly absent in the *Chd1* mutant flies. Complementary PCR-based assays showed a loss of CHD1 correlated with an accumulation of *Acetobacter* and a decrease in *Lactobacillus* species. These effects were age dependent and more pronounced in younger flies. Importantly, the authors showed that *CHD1*^−/−^ flies were unable to sustain *L. plantarum* titres after dietary supplementation. Future research needs to determine to what extent gene regulation relevant to microbe-host interactions is the direct result of chromatin remodelling by CHD1 over the genes as opposed to some indirect effects. It will also be very exciting to discover if the role of CHD1 in host-microbe interactions is conserved in mammals.

## 11 Outlook

The microbiota affect gene regulation of the intestinal epithelium in various ways, of which the generation of SCFA is a dominant pathway. Inhibition of HDACs by SCFA is an important mechanism. As SCFA also are an important energy source in the gut, future studies need to elucidate to what extent SCFA affect chromatin by providing metabolic precursors in the cell, for example, butyryl-CoA, for mediating alternative histone acylations.

Microbiota-host interactions are fascinating and important to study. However, this field poses many challenges [81]. While we presented several examples in this review, in which the deletion of chromatin factors affected host–microbiome interactions, the extent to which the microbiome is affected by genetic variations in the general population is an area of debate and intense research [23,82]. A considerable problem in studying microbiota-host interactions is the fact that the microbiota is highly dynamic and diverse. Therefore, mice in various facilities, even specific or pathogen-free (SOPF), differ markedly in their microbiota, resulting, in different experimental colitis outcomes (see for example [83]). Furthermore, mice in clean SOPF facilities have reduced microbiota, with consequences to their immune system and physiology [[84], [85], [86]]. Therefore, future studies should consider the normal rich “healthy” microbiota of wild mice. These problems are even more challenging considering the human microbiome where greater diversity in genetic background, lifestyle, and other factors further complicate studies of the interactions between hosts and microbiota.

While this report focused on gut microbiota, mucosal surfaces in other tissues are covered with their specific microbiota. For example, the uterus has a microbiota that affects pregnancy outcomes [87]. Inter-kingdom crosstalk is important in all of these compartments, and regulation through chromatin dynamics is likely also going to be an important facet. We are only beginning to understand the mechanisms of microbiota-host interactions, many of which have been “hard-wired” into our genome through co-evolution. In the future, more aspects of chromatin dynamics are likely to be revealed as essential in this process.

## References

[1] Castillo J., López-Rodas G., Franco L. (2017). Histone post-translational modifications and nucleosome organisation in transcriptional regulation: some open questions. Advances in Experimental Medicine and Biology.

[2] Suganuma T., Workman J.L. (2011). Signals and combinatorial functions of histone modifications. Annual Review of Biochemistry.

[3] Yun M., Wu J., Workman J.L., Li B. (2011). Readers of histone modifications. Cell Research.

[4] Hyun K., Jeon J., Park K., Kim J. (2017). Writing, erasing and reading histone lysine methylations. Experimental and Molecular Medicine.

[5] Varga-Weisz P.D. (2014). Chromatin remodeling: a collaborative effort. Nature Structural and Molecular Biology.

[6] Clapier C.R., Cairns B.R. (2009). The biology of chromatin remodeling complexes. Annual Review of Biochemistry.

[7] Cavalli G., Heard E. (2019). Advances in epigenetics link genetics to the environment and disease. Nature.

[8] Qin Y., Wade P.A. (2018). Crosstalk between the microbiome and epigenome: messages from bugs. Journal of Biochemistry.

[9] Krautkramer K.A., Rey F.E., Denu J.M. (2017). Chemical signaling between gut microbiota and host chromatin: what is your gut really saying?. Journal of Biological Chemistry.

[10] Miro-Blanch J., Yanes O. (2019). Epigenetic regulation at the interplay between gut microbiota and host metabolism. Frontiers in Genetics.

[11] Allen J., Sears C.L. (2019). Impact of the gut microbiome on the genome and epigenome of colon epithelial cells: contributions to colorectal cancer development. Genome Medicine.

[12] Riscuta G., Xi D., Pierre-Victor D., Starke-Reed P., Khalsa J., Duffy L. (2018). Diet, microbiome, and epigenetics in the era of precision medicine. Methods in Molecular Biology.

[13] Ye J., Wu W., Li Y., Li L. (2017). Influences of the gut microbiota on DNA methylation and histone modification. Digestive Diseases and Sciences.

[14] Woo V., Alenghat T. (2017). Host-microbiota interactions: epigenomic regulation. Current Opinion in Immunology.

[15] Lloyd-Price J., Mahurkar A., Rahnavard G., Crabtree J., Orvis J., Hall A.B. (2017). Strains, functions and dynamics in the expanded human microbiome project. Nature.

[16] Pasolli E., Asnicar F., Manara S., Zolfo M., Karcher N., Armanini F. (2019). Extensive unexplored human microbiome diversity revealed by over 150,000 genomes from metagenomes spanning age, geography, and lifestyle. Cell.

[17] Ding T., Schloss P.D. (2014). Dynamics and associations of microbial community types across the human body. Nature.

[18] Integrative HMP (iHMP) Research Network Consortium (2019). The integrative human microbiome project. Nature.

[19] Foster K.R., Schluter J., Coyte K.Z., Rakoff-Nahoum S. (2017). The evolution of the host microbiome as an ecosystem on a leash. Nature.

[20] Sender R., Fuchs S., Milo R. (2016). Are we really vastly outnumbered? Revisiting the ratio of bacterial to host cells in humans. Cell.

[21] Donohoe D.R., Garge N., Zhang X., Sun W., O'Connell T.M., Bunger M.K. (2011). The microbiome and butyrate regulate energy metabolism and autophagy in the mammalian colon. Cell Metabolism.

[22] Kennedy E.A., King K.Y., Baldridge M.T. (2018). Mouse microbiota models: comparing germ-free mice and antibiotics treatment as tools for modifying gut bacteria. Frontiers in Physiology.

[23] Rothschild D., Weissbrod O., Barkan E., Kurilshikov A., Korem T., Zeevi D. (2018). Environment dominates over host genetics in shaping human gut microbiota. Nature.

[24] Kundu P., Blacher E., Elinav E., Pettersson S. (2017). Our gut microbiome: the evolving inner self. Cell.

[25] Rivera-Chávez F., Bäumler A.J. (2015). The pyromaniac inside you: Salmonella metabolism in the host gut. Annual Review of Microbiology.

[26] Plichta D.R., Graham D.B., Subramanian S., Xavier R.J. (2019). Therapeutic opportunities in inflammatory bowel disease: mechanistic dissection of host-microbiome relationships. Cell.

[27] Marshall B.J., Warren J.R. (1984). Unidentified curved bacilli in the stomach of patients with gastritis and peptic ulceration. Lancet.

[28] Peterson L.W., Artis D. (2014). Intestinal epithelial cells: regulators of barrier function and immune homeostasis. Nature Reviews Immunology.

[29] Rooks M.G., Garrett W.S. (2016). Gut microbiota, metabolites and host immunity. Nature Reviews Immunology.

[30] Li Y., Innocentin S., Withers D.R., Roberts N.A., Gallagher A.R., Grigorieva E.F. (2011). Exogenous stimuli maintain intraepithelial lymphocytes via aryl hydrocarbon receptor activation. Cell.

[31] Zelante T., Iannitti R.G., Cunha C., De Luca A., Giovannini G., Pieraccini G. (2013). Tryptophan catabolites from microbiota engage aryl hydrocarbon receptor and balance mucosal reactivity via interleukin-22. Immunity.

[32] Moura-Alves P., Faé K., Houthuys E., Dorhoi A., Kreuchwig A., Furkert J. (2014). AhR sensing of bacterial pigments regulates antibacterial defence. Nature.

[33] LeBlanc J.G., Milani C., de Giori G.S., Sesma F., van Sinderen D., Ventura M. (2013). Bacteria as vitamin suppliers to their host: a gut microbiota perspective. Current Opinion in Biotechnology.

[34] Crider K.S., Yang T.P., Berry R.J., Bailey L.B. (2012). Folate and DNA methylation: a review of molecular mechanisms and the evidence for folate's role. Advance in Nutrition.

[35] Kok D.E., Steegenga W.T., McKay J.A. (2018). Folate and epigenetics: why we should not forget bacterial biosynthesis. Epigenomics.

[36] Tofalo R., Cocchi S., Suzzi G. (2019). Polyamines and gut microbiota. Frontiers in Nutrition.

[37] Koh A., De Vadder F., Kovatcheva-Datchary P., Bäckhed F. (2016). From dietary fiber to host physiology: short-chain fatty acids as key bacterial metabolites. Cell.

[38] Rombeau J.L., Kripke S.A., Settle R.G., Kritchevsky D., Bonfield C., Anderson J.W. (1990). last. Short-chain fatty acids. Production, absorption, metabolism, and intestinal effects. Dietary Fiber.

[39] Ferreira-Halder C.V., de S Faria A.V., Andrade S.S. (2017). Action and function of Faecalibacterium prausnitzii in health and disease. Best Practice and Research Clinical Gastroenterology.

[40] Zarrinpar A., Chaix A., Xu Z.Z., Chang M.W., Marotz C.A., Saghatelian A. (2018). Antibiotic-induced microbiome depletion alters metabolic homeostasis by affecting gut signaling and colonic metabolism. Nature Communications.

[41] Kelly C.J., Zheng L., Campbell E.L., Saeedi B., Scholz C.C., Bayless A.J. (2015). Crosstalk between microbiota-derived short-chain fatty acids and intestinal epithelial HIF augments tissue barrier function. Cell Host and Microbe.

[42] Fachi J.L., de S Felipe J., Pral L.P., da Silva B.K., Corrêa R.O., de Andrade M.C.P. (2019). Butyrate protects mice from Clostridium difficile-induced colitis through an HIF-1-Dependent mechanism. Cell Reports.

[43] Kaiko G.E., Ryu S.H., Koues O.I., Collins P.L., Solnica-Krezel L., Pearce E.J. (2016). The colonic crypt protects stem cells from microbiota-derived metabolites. Cell.

[44] Carrer A., Parris J.L.D., Trefely S., Henry R.A., Montgomery D.C., Torres A. (2017). Impact of a high-fat diet on tissue acyl-CoA and histone acetylation levels. Journal of Biological Chemistry.

[45] Su X., Wellen K.E., Rabinowitz J.D. (2016). Metabolic control of methylation and acetylation. Current Opinion in Chemical Biology.

[46] Boffa L.C., Lupton J.R., Mariani M.R., Ceppi M., Newmark H.L., Scalmati A. (1992). Modulation of colonic epithelial cell proliferation, histone acetylation, and luminal short chain fatty acids by variation of dietary fiber (wheat bran) in rats. Cancer Research.

[47] Krautkramer K.A., Kreznar J.H., Romano K.A., Vivas E.I., Barrett-Wilt G.A., Rabaglia M.E. (2016). Diet-microbiota interactions mediate global epigenetic programming in multiple host tissues. Molecular Cell.

[48] Smith K., McCoy K.D., Macpherson A.J. (2007). Use of axenic animals in studying the adaptation of mammals to their commensal intestinal microbiota. Seminars in Immunology.

[49] Wellen K.E., Hatzivassiliou G., Sachdeva U.M., Bui T.V., Cross J.R., Thompson C.B. (2009). ATP-citrate lyase links cellular metabolism to histone acetylation. Science.

[50] Sabari B.R., Zhang D., Allis C.D., Zhao Y. (2017). Metabolic regulation of gene expression through histone acylations. Nature Reviews Molecular Cell Biology.

[51] Kebede A.F., Nieborak A., Shahidian L.Z., Le Gras S., Richter F., Gómez D.A. (2017). Histone propionylation is a mark of active chromatin. Nature Structural and Molecular Biology.

[52] Zhao S., Zhang X., Li H. (2018). Beyond histone acetylation-writing and erasing histone acylations. Current Opinion in Structural Biology.

[53] Sabari B.R., Tang Z., Huang H., Yong-Gonzalez V., Molina H., Kong H.E. (2018). Intracellular crotonyl-CoA stimulates transcription through p300-catalyzed histone crotonylation. Molecular Cell.

[54] Andrews F.H., Shinsky S.A., Shanle E.K., Bridgers J.B., Gest A., Tsun I.K. (2016). The Taf14 YEATS domain is a reader of histone crotonylation. Nature Chemical Biology.

[55] Li Y., Sabari B.R., Panchenko T., Wen H., Zhao D., Guan H. (2016). Molecular coupling of histone crotonylation and active transcription by AF9 YEATS domain. Molecular Cell.

[56] Zhang Q., Zeng L., Zhao C., Ju Y., Konuma T., Zhou M.-M. (2016). Structural insights into histone crotonyl-lysine recognition by the AF9 YEATS domain. Structure.

[57] Fellows R., Denizot J., Stellato C., Cuomo A., Jain P., Stoyanova E. (2018). Microbiota derived short chain fatty acids promote histone crotonylation in the colon through histone deacetylases. Nature Communications.

[58] Wei W., Liu X., Chen J., Gao S., Lu L., Zhang H. (2017). Class I histone deacetylases are major histone decrotonylases: evidence for critical and broad function of histone crotonylation in transcription. Cell Research.

[59] Kelly R.D.W., Chandru A., Watson P.J., Song Y., Blades M., Robertson N.S. (2018). Histone deacetylase (HDAC) 1 and 2 complexes regulate both histone acetylation and crotonylation in vivo. Scientific Reports.

[60] Alenghat T., Osborne L.C., Saenz S.A., Kobuley D., Ziegler C.G.K., Mullican S.E. (2013). Histone deacetylase 3 coordinates commensal-bacteria-dependent intestinal homeostasis. Nature.

[61] Adolph T.E., Mayr L., Grabherr F., Tilg H. (2018). Paneth cells and their antimicrobials in intestinal immunity. Current Pharmaceutical Design.

[62] Navabi N., Whitt J., Wu S.-E., Woo V., Moncivaiz J., Jordan M.B. (2017). Epithelial histone deacetylase 3 instructs intestinal immunity by coordinating local lymphocyte activation. Cell Reports.

[63] Wellman A.S., Metukuri M.R., Kazgan N., Xu X., Xu Q., Ren N.S.X. (2017). Intestinal epithelial sirtuin 1 regulates intestinal inflammation during aging in mice by altering the intestinal microbiota. Gastroenterology.

[64] Lo Sasso G., Ryu D., Mouchiroud L., Fernando S.C., Anderson C.L., Katsyuba E. (2014). Loss of Sirt1 function improves intestinal anti-bacterial defense and protects from colitis-induced colorectal cancer. PLoS One.

[65] Eskandarian H.A., Impens F., Nahori M.-A., Soubigou G., Coppée J.-Y., Cossart P. (2013). A role for SIRT2-dependent histone H3K18 deacetylation in bacterial infection. Science.

[66] Pereira J.M., Chevalier C., Chaze T., Gianetto Q., Impens F., Matondo M. (2018). Infection reveals a modification of SIRT2 critical for chromatin association. Cell Reports.

[67] Zhang X., Cao R., Niu J., Yang S., Zhao S., Li H. (2018). Molecular basis for hierarchical histone de-Β-hydroxybutyrylation by Sirt3. SSRN Electronic Journal.

[68] Yue F., Cheng Y., Breschi A., Vierstra J., Wu W., Ryba T. (2014). A comparative encyclopedia of DNA elements in the mouse genome. Nature.

[69] Qin Y., Roberts J.D., Grimm S.A., Lih F.B., Deterding L.J., Li R. (2018). An obesity-associated gut microbiome reprograms the intestinal epigenome and leads to altered colonic gene expression. Genome Biology.

[70] Cooke J., Zhang H., Greger L., Silva A.-L., Massey D., Dawson C. (2012). Mucosal genome-wide methylation changes in inflammatory bowel disease. Inflammatory Bowel Diseases.

[71] Ventham N.T., Kennedy N.A., Adams A.T., Kalla R., Heath S., O'Leary K.R. (2016). Integrative epigenome-wide analysis demonstrates that DNA methylation may mediate genetic risk in inflammatory bowel disease. Nature Communications.

[72] McDermott E., Ryan E.J., Tosetto M., Gibson D., Burrage J., Keegan D. (2016). DNA methylation profiling in inflammatory bowel disease provides new insights into disease pathogenesis. Journal Crohns Colitis.

[73] Harris R.A., Shah R., Hollister E.B., Tronstad R.R., Hovdenak N., Szigeti R. (2016). Colonic mucosal epigenome and microbiome development in children and adolescents. Journal of Immunology Research.

[74] Lin Z., Hegarty J., Cappel J., Yu W., Chen X., Faber P. (2011). Identification of disease-associated DNA methylation in intestinal tissues from patients with inflammatory bowel disease. Clinical Genetics.

[75] Kelly D., Kotliar M., Woo V., Jagannathan S., Whitt J., Moncivaiz J. (2018). Microbiota-sensitive epigenetic signature predicts inflammation in Crohn's disease. JCI Insight.

[76] Chen K., Luan X., Liu Q., Wang J., Chang X., Snijders A.M. (2019). Drosophila histone demethylase KDM5 regulates social behavior through immune control and gut microbiota maintenance. Cell Host and Microbe.

[77] Farrelly L.A., Thompson R.E., Zhao S., Lepack A.E., Lyu Y., Bhanu N.V. (2019). Histone serotonylation is a permissive modification that enhances TFIID binding to H3K4me3. Nature.

[78] Konev A.Y., Tribus M., Park S.Y., Podhraski V., Lim C.Y., Emelyanov A.V. (2007). CHD1 motor protein is required for deposition of histone variant H3.3 into chromatin in vivo. Science.

[79] Sebald J., Morettini S., Podhraski V., Lass-Flörl C., Lusser A. (2012). CHD1 contributes to intestinal resistance against infection by P. aeruginosa in Drosophila melanogaster. PLoS One.

[80] Sebald J., Willi M., Schoberleitner I., Krogsdam A., Orth-Höller D., Trajanoski Z. (2016). Impact of the chromatin remodeling factor CHD1 on gut microbiome composition of Drosophila melanogaster. PLoS One.

[81] Goodrich J.K., Di Rienzi S.C., Poole A.C., Koren O., Walters W.A., Caporaso J.G. (2014). Conducting a microbiome study. Cell.

[82] Goodrich J.K., Waters J.L., Poole A.C., Sutter J.L., Koren O., Blekhman R. (2014). Human genetics shape the gut microbiome. Cell.

[83] Roy U., Gálvez E.J.C., Iljazovic A., Lesker T.R., Błażejewski A.J., Pils M.C. (2017). Distinct microbial communities trigger colitis development upon intestinal barrier damage via innate or adaptive immune cells. Cell Reports.

[84] Beura L.K., Hamilton S.E., Bi K., Schenkel J.M., Odumade O.A., Casey K.A. (2016). Normalizing the environment recapitulates adult human immune traits in laboratory mice. Nature.

[85] Rosshart S.P., Herz J., Vassallo B.G., Hunter A., Wall M.K., Badger J.H. (2019). Laboratory mice born to wild mice have natural microbiota and model human immune responses. Science.

[86] Rosshart S.P., Vassallo B.G., Angeletti D., Hutchinson D.S., Morgan A.P., Takeda K. (2017). Wild mouse gut microbiota promotes host fitness and improves disease resistance. Cell.

[87] Heil B.A., Paccamonti D.L., Sones J.L. (2019). Role for the mammalian female reproductive tract microbiome in pregnancy outcomes. Physiological Genomics.

[88] Li Y., Seto E. (2016). HDACs and HDAC inhibitors in cancer development and therapy. Cold Spring in Harbour Perspective Medicine.

[89] Jenuwein T., Allis C.D. (2001). Translating the histone code. Science.

[90] Clevers H., Batlle E. (2013). SnapShot: the intestinal crypt. Cell.

[91] Hansson G.C. (2012). Role of mucus layers in gut infection and inflammation. Current Opinion in Microbiology.

[92] Rao J.N., Wang J.-Y. (2010). Regulation of gastrointestinal mucosal growth [Internet]. http://www.ncbi.nlm.nih.gov/books/NBK54091/.

[93] Wang J., Thingholm L.B., Skiecevičienė J., Rausch P., Kummen M., Hov J.R. (2016). Genome-wide association analysis identifies variation in vitamin D receptor and other host factors influencing the gut microbiota. Nature Genetics.

[94] Wostmann B.S. (1981). The germfree animal in nutritional studies. Annual Review of Nutrition.

[95] Sumi Y., Miyakawa M., Kanzaki M., Kotake Y. (1977). Vitamin B-6 deficiency in germfree rats. Journal of Nutrition.

[96] Yoshii K., Hosomi K., Sawane K., Kunisawa J. (2019). Metabolism of dietary and microbial vitamin B family in the regulation of host immunity. Frontiers in Nutrition.

[97] Magnśsdóttir S., Ravcheev D., de Crécy-Lagard V., Thiele I. (2015). Systematic genome assessment of B-vitamin biosynthesis suggests co-operation among gut microbes. Frontiers in Genetics.

[98] Suttie J.W. (1995). The importance of menaquinones in human nutrition. Annual Review of Nutrition.

[99] Connors J., Dawe N., Van Limbergen J. (2018). The role of succinate in the regulation of intestinal inflammation. Nutrients.

[100] Ridlon J.M., Kang D.J., Hylemon P.B., Bajaj J.S. (2014). Bile acids and the gut microbiome. Current Opinion in Gastroenterology.

[101] Nicholson J.K., Wilson I.D. (2003). Opinion: understanding “global” systems biology: metabonomics and the continuum of metabolism. Nature Reviews Drug Discovery.

[102] Natividad J.M., Agus A., Planchais J., Lamas B., Jarry A.C., Martin R. (2018). Impaired aryl hydrocarbon receptor ligand production by the gut microbiota is a key factor in metabolic syndrome. Cell Metabolism.

[103] Ozdal T., Sela D.A., Xiao J., Boyacioglu D., Chen F., Capanoglu E. (2016). The reciprocal interactions between polyphenols and gut microbiota and effects on bioaccessibility. Nutrients.

[104] Rajavelu A., Tulyasheva Z., Jaiswal R., Jeltsch A., Kuhnert N. (2011). The inhibition of the mammalian DNA methyltransferase 3a (Dnmt3a) by dietary black tea and coffee polyphenols. BMC Biochemistry.

[105] Nandakumar V., Vaid M., Katiyar S.K. (2011). (-)-Epigallocatechin-3-gallate reactivates silenced tumor suppressor genes, Cip1/p21 and p16INK4a, by reducing DNA methylation and increasing histones acetylation in human skin cancer cells. Carcinogenesis.

[106] Vahid F., Zand H., Nosrat-Mirshekarlou E., Najafi R., Hekmatdoost A. (2015). The role dietary of bioactive compounds on the regulation of histone acetylases and deacetylases: a review. Gene.

[107] Takagaki A., Nanjo F. (2013). Catabolism of (+)-catechin and (-)-epicatechin by rat intestinal microbiota. Journal of Agricultural and Food Chemistry.

[108] Winter J., Moore L.H., Dowell V.R., Bokkenheuser V.D. (1989). C-ring cleavage of flavonoids by human intestinal bacteria. Applied and Environmental Microbiology.

[109] Miyamoto J., Igarashi M., Watanabe K., Karaki S.-I., Mukouyama H., Kishino S. (2019). Gut microbiota confers host resistance to obesity by metabolizing dietary polyunsaturated fatty acids. Nature Communications.

[110] Miyamoto J., Mizukure T., Park S.-B., Kishino S., Kimura I., Hirano K. (2015). A gut microbial metabolite of linoleic acid, 10-hydroxy-cis-12-octadecenoic acid, ameliorates intestinal epithelial barrier impairment partially via GPR40-MEK-ERK pathway. Journal of Biological Chemistry.

[111] Kishino S., Takeuchi M., Park S.-B., Hirata A., Kitamura N., Kunisawa J. (2013). Polyunsaturated fatty acid saturation by gut lactic acid bacteria affecting host lipid composition. Proceedings of the National Academy of Sciences of the United States of America.

[112] Nanthirudjanar T., Furumoto H., Zheng J., Kim Y.-I., Goto T., Takahashi N. (2015). Gut microbial fatty acid metabolites reduce triacylglycerol levels in hepatocytes. Lipids.

[113] Ohue-Kitano R., Yasuoka Y., Goto T., Kitamura N., Park S.-B., Kishino S. (2018). α-Linolenic acid-derived metabolites from gut lactic acid bacteria induce differentiation of anti-inflammatory M2 macrophages through G protein-coupled receptor 40. The FASEB Journal.

[114] Dimri M., Bommi P.V., Sahasrabuddhe A.A., Khandekar J.D., Dimri G.P. (2010). Dietary omega-3 polyunsaturated fatty acids suppress expression of EZH2 in breast cancer cells. Carcinogenesis.

[115] Dumas M.-E., Barton R.H., Toye A., Cloarec O., Blancher C., Rothwell A. (2006). Metabolic profiling reveals a contribution of gut microbiota to fatty liver phenotype in insulin-resistant mice. Proceedings of the National Academy of Sciences of the United States of America.

[116] Kaelin W.G., McKnight S.L. (2013). Influence of metabolism on epigenetics and disease. Cell.

[117] Xiao M., Yang H., Xu W., Ma S., Lin H., Zhu H. (2012). Inhibition of α-KG-dependent histone and DNA demethylases by fumarate and succinate that are accumulated in mutations of FH and SDH tumor suppressors. Genes and Development.

[118] Rath S., Heidrich B., Pieper D.H., Vital M. (2017). Uncovering the trimethylamine-producing bacteria of the human gut microbiota. Microbiome.

[119] Nag A., St John P.C., Crowley M.F., Bomble Y.J. (2018). Prediction of reaction knockouts to maximize succinate production by Actinobacillus succinogenes. PLoS One.

[120] Serena C., Ceperuelo-Mallafré V., Keiran N., Queipo-Ortuño M.I., Bernal R., Gomez-Huelgas R. (2018). Elevated circulating levels of succinate in human obesity are linked to specific gut microbiota. The ISME Journal.

[121] Louis P., Hold G.L., Flint H.J. (2014). The gut microbiota, bacterial metabolites and colorectal cancer. Nature Reviews Microbiology.

[122] Abdul Rahim M.B.H., Chilloux J., Martinez-Gili L., Neves A.L., Myridakis A., Gooderham N. (2019). Diet-induced metabolic changes of the human gut microbiome: importance of short-chain fatty acids, methylamines and indoles. Acta Diabetologica.

[123] Samuel B.S., Shaito A., Motoike T., Rey F.E., Backhed F., Manchester J.K. (2008). Effects of the gut microbiota on host adiposity are modulated by the short-chain fatty-acid binding G protein-coupled receptor, Gpr41. Proceedings of the National Academy of Sciences of the United States of America.

[124] Cousens L.S., Gallwitz D., Alberts B.M. (1979). Different accessibilities in chromatin to histone acetylase. Journal of Biological Chemistry.

[125] Reichardt N., Duncan S.H., Young P., Belenguer A., McWilliam Leitch C., Scott K.P. (2014). Phylogenetic distribution of three pathways for propionate production within the human gut microbiota. The ISME Journal.

[126] Perry R.J., Peng L., Barry N.A., Cline G.W., Zhang D., Cardone R.L. (2016). Acetate mediates a microbiome-brain-β-cell axis to promote metabolic syndrome. Nature.

[127] Fukuda S., Toh H., Taylor T.D., Ohno H., Hattori M. (2012). Acetate-producing bifidobacteria protect the host from enteropathogenic infection via carbohydrate transporters. Gut Microbes.

[128] Feng W., Ao H., Peng C. (2018). Gut microbiota, short-chain fatty acids, and herbal medicines. Frontiers in Pharmacology.

[129] Louis P., Flint H.J. (2009). Diversity, metabolism and microbial ecology of butyrate-producing bacteria from the human large intestine. FEMS Microbiology Letters.

[130] Walker A.W., Duncan S.H., McWilliam Leitch E.C., Child M.W., Flint H.J. (2005). pH and peptide supply can radically alter bacterial populations and short-chain fatty acid ratios within microbial communities from the human colon. Applied and Environmental Microbiology.

[131] Musso G., Gambino R., Cassader M. (2011). Interactions between gut microbiota and host metabolism predisposing to obesity and diabetes. Annual Review of Medicine.

[132] Chang P.V., Hao L., Offermanns S., Medzhitov R. (2014). The microbial metabolite butyrate regulates intestinal macrophage function via histone deacetylase inhibition. Proceedings of the National Academy of Sciences of the United States of America.

[133] Nicholson J.K., Holmes E., Kinross J., Burcelin R., Gibson G., Jia W. (2012). Host-gut microbiota metabolic interactions. Science.

[134] Levy M., Kolodziejczyk A.A., Thaiss C.A., Elinav E. (2017). Dysbiosis and the immune system. Nature Reviews Immunology.

[135] Berndsen C.E., Denu J.M. (2008). Catalysis and substrate selection by histone/protein lysine acetyltransferases. Current Opinion in Structural Biology.

[136] Lange M., Kaynak B., Forster U.B., Tönjes M., Fischer J.J., Grimm C. (2008). Regulation of muscle development by DPF3, a novel histone acetylation and methylation reader of the BAF chromatin remodeling complex. Genes and Development.

[137] Filippakopoulos P., Knapp S. (2014). Targeting bromodomains: epigenetic readers of lysine acetylation. Nature Reviews Drug Discovery.

[138] Li Y., Wen H., Xi Y., Tanaka K., Wang H., Peng D. (2014). AF9 YEATS domain links histone acetylation to DOT1L-mediated H3K79 methylation. Cell.

[139] Narita T., Weinert B.T., Choudhary C. (2019). Functions and mechanisms of non-histone protein acetylation. Nature Reviews Molecular Cell Biology.

[140] Chen Y., Sprung R., Tang Y., Ball H., Sangras B., Kim S.C. (2007). Lysine propionylation and butyrylation are novel post-translational modifications in histones. Molecular and Cellular Proteomics.

[141] Montgomery D.C., Sorum A.W., Meier J.L. (2015). Defining the orphan functions of lysine acetyltransferases. ACS Chemical Biology.

[142] Han Z., Wu H., Kim S., Yang X., Li Q., Huang H. (2018). Revealing the protein propionylation activity of the histone acetyltransferase MOF (males absent on the first). Journal of Biological Chemistry.

[143] Leemhuis H., Packman L.C., Nightingale K.P., Hollfelder F. (2008). The human histone acetyltransferase P/CAF is a promiscuous histone propionyltransferase. ChemBioChem.

[144] Kaczmarska Z., Ortega E., Goudarzi A., Huang H., Kim S., Márquez J.A. (2017). Structure of p300 in complex with acyl-CoA variants. Nature Chemical Biology.

[145] Flynn E.M., Huang O.W., Poy F., Oppikofer M., Bellon S.F., Tang Y. (2015). A subset of human bromodomains recognizes butyryllysine and crotonyllysine histone peptide modifications. Structure.

[146] Xiong X., Panchenko T., Yang S., Zhao S., Yan P., Zhang W. (2016). Selective recognition of histone crotonylation by double PHD fingers of MOZ and DPF2. Nature Chemical Biology.

[147] Feldman J.L., Baeza J., Denu J.M. (2013). Activation of the protein deacetylase SIRT6 by long-chain fatty acids and widespread deacylation by mammalian sirtuins. Journal of Biological Chemistry.

[148] Sabari B.R., Tang Z., Huang H., Yong-Gonzalez V., Molina H., Kong H.E. (2015). Intracellular crotonyl-CoA stimulates transcription through p300-catalyzed histone crotonylation. Molecular Cell.

[149] Liu X., Wei W., Liu Y., Yang X., Wu J., Zhang Y. (2017). MOF as an evolutionarily conserved histone crotonyltransferase and transcriptional activation by histone acetyltransferase-deficient and crotonyltransferase-competent CBP/p300. Cellular Discovery.

[150] Zhao D., Guan H., Zhao S., Mi W., Wen H., Li Y. (2016). YEATS2 is a selective histone crotonylation reader. Cell Research.

[151] Bao X., Wang Y., Li X., Li X.-M., Liu Z., Yang T. (2014). Identification of “erasers” for lysine crotonylated histone marks using a chemical proteomics approach. Elife.

[152] Madsen A.S., Olsen C.A. (2012). Profiling of substrates for zinc-dependent lysine deacylase enzymes: HDAC3 exhibits decrotonylase activity in vitro. Angewandte Chemie International Edition in English.

